# Covalent Aurora A regulation by the metabolic integrator coenzyme A

**DOI:** 10.1016/j.redox.2019.101318

**Published:** 2019-09-05

**Authors:** Yugo Tsuchiya, Dominic P. Byrne, Selena G. Burgess, Jenny Bormann, Jovana Baković, Yueyang Huang, Alexander Zhyvoloup, Bess Yi Kun Yu, Sew Peak-Chew, Trang Tran, Fiona Bellany, Alethea B. Tabor, AW Edith Chan, Lalitha Guruprasad, Oleg Garifulin, Valeriy Filonenko, Matthias Vonderach, Samantha Ferries, Claire E. Eyers, John Carroll, Mark Skehel, Richard Bayliss, Patrick A. Eyers, Ivan Gout

**Affiliations:** a Department of Structural and Molecular Biology, University College London, London, WC1E 6BT, UK; b Department of Biochemistry, Institute of Integrative Biology, University of Liverpool, Liverpool, L69 7ZB, UK; c School of Molecular and Cellular Biology, Astbury Centre for Structural and Molecular Biology, University of Leeds, Leeds, LS2 9JT, UK; d Department of Cell and Developmental Biology, University College London, London, WC1E 6BT, UK; e MRC Laboratory of Molecular Biology, Cambridge Biomedical Campus, Cambridge, CB2 0QH, UK; f Department of Chemistry, University College London, London, WC1E 6BT, UK; g Wolfson Institute for Biomedical Research, University College London, London, WC1E 6BT, UK; h School of Chemistry, University of Hyderabad, Hyderabad, 500 046, India; i Department of Cell Signaling, Institute of Molecular Biology and Genetics, Kyiv 143, Ukraine; j Centre for Proteome Research, Department of Biochemistry, Institute of Integrative Biology, University of Liverpool, Liverpool, L69 7ZB, UK

## Abstract

Aurora A kinase is a master mitotic regulator whose functions are controlled by several regulatory interactions and post-translational modifications. It is frequently dysregulated in cancer, making Aurora A inhibition a very attractive antitumor target. However, recently uncovered links between Aurora A, cellular metabolism and redox regulation are not well understood. In this study, we report a novel mechanism of Aurora A regulation in the cellular response to oxidative stress through CoAlation. A combination of biochemical, biophysical, crystallographic and cell biology approaches revealed a new and, to our knowledge, unique mode of Aurora A inhibition by CoA, involving selective binding of the ADP moiety of CoA to the ATP binding pocket and covalent modification of Cys290 in the activation loop by the thiol group of the pantetheine tail. We provide evidence that covalent CoA modification (CoAlation) of Aurora A is specific, and that it can be induced by oxidative stress in human cells. Oxidising agents, such as diamide, hydrogen peroxide and menadione were found to induce Thr 288 phosphorylation and DTT-dependent dimerization of Aurora A. Moreover, microinjection of CoA into fertilized mouse embryos disrupts bipolar spindle formation and the alignment of chromosomes, consistent with Aurora A inhibition.

Altogether, our data reveal CoA as a new, rather selective, inhibitor of Aurora A, which locks this kinase in an inactive state via a “dual anchor” mechanism of inhibition that might also operate in cellular response to oxidative stress. Finally and most importantly, we believe that these novel findings provide a new rationale for developing effective and irreversible inhibitors of Aurora A, and perhaps other protein kinases containing appropriately conserved Cys residues.

## 1 Introduction

Aurora kinases are Ser/Thr kinases that play well-documented roles in eukaryotes, where they control meiosis, mitosis and cell division [1]. Aurora kinases differ in relative expression levels, stability and sub-cellular localization, the latter linked to single amino acid differences that dramatically affect biological distribution and function [[2], [3], [4]]. In vertebrates, the Aurora family of protein kinases are grouped into two distinct sub-families composed of Aurora A and Aurora B and C [5]. Aurora kinases contain a conserved C-terminal kinase domain, which phosphorylates substrates with a minimal R-X-S/T consensus motif [6,7]. The Aurora kinase domain is itself regulated by a critical autophosphorylation event in the activation loop [[8], [9], [10], [11]], which is associated with catalytic activation and timely execution of different phases of mitosis [[12], [13], [14], [15]] and meiosis [16]. During the cell cycle, Aurora A has canonical roles in centrosome duplication, spindle bipolarity, chromosome segregation and spindle checkpoint maintenance [17], which together contribute to fundamental processes such as centrosomal and genomic stability and likely reflect the long-recognised oncogenic properties associated with overexpressed Aurora A [18]. Interestingly, Aurora A has also recently been implicated in the control of energy production in cancer cells through mitochondrial targeting [19]. In contrast, Aurora B is a chromosomal passenger protein required for the spindle assembly checkpoint and rate-limiting for cytokinesis in cells [20]. Aurora C is most highly expressed in germ cells, where it can functionally replace Auora B as a chromosome passenger protein [1].

Aurora A biology is controlled *via* targeted subcellular localization mediated through the formation of dynamic protein complexes with non-catalytic binding partners such as TPX2 [9,10,[21], [22], [23]], TACC3 [24] and NMYC [25], which control distinct Aurora A signaling outputs. Indeed, Aurora A/TPX2 holoenzyme complexes co-localise at the polar end of spindle microtubules, where Aurora A is maintained in dynamic pools alongside the mitoitc kinesin Eg5, which is localised by a C-terminal motif in TPX2 [22,26,27]. Allosteric Aurora A activation on the spindle is thought to occur by binding to microtubule-associated proteins (which are also Aurora A substrates) such as TPX2 and TACC3 [28]. Mechanistically, Aurora A activation requires autophosphorylation of Thr 288 (and possibly Thr 287), which drives the kinase into the most catalytically-competent conformation [3,9,29]. Interestingly, the TPX2 complex also protects Aurora A from Thr 288 dephosphorylation by PP1 and PP2A phosphatases *in vitro* [3,9], although accumulating evidence suggests that the PP6 Ser/Thr phosphatase is rate-limiting for mitotic dephosphorylation at this site in cells [27]. In addition to TPX2, Aurora A is targeted to core protein co-factors, including the centrosomal protein Cep192, the mitotic entry factor BORA and the transcription factor NMYC [30], whose interaction with a specific inactive Aurora A conformation is important for controlling NMYC stability in cells, and the basis for new approaches to target Aurora A output with drugs [30].

Structural analysis of Aurora A confirms the importance of activation loop dynamics for activity and binding-partner interactions [24]. Phosphorylation, as well as TPX2 binding, stabilise the activation segement in an appropriate conformation for catalysis, and distinct Aurora A conformations can also be induced and/or stabilised by a huge variety of chemical small molecules [13,14,[31], [32], [33], [34]]. Such compounds have been extremely useful to validate dozens of cellular Aurora A substrates [6,13], which include cell cycle-regulated kinases such as PLK1 [14,35].

In a clinical context, overexpression of Aurora A is frequently detected in human malignancies, including leukemia, breast, prostate and colon cancers [18,36,37], and lower overall survival in patients with colorectal cancer correlates with increased levels of Aurora A [38]. For these reasons, Aurora A has been pursued for decades as a target for the development of anti-cancer therapeutic agents, some of which show potential in the clinic. Beginning with MLN8054 [39], a number of potent, and highly selective, ATP-competitive small molecule inhibitors of Aurora A have been reported, with several advancing to phase II clinical trials, and MLN8237 (alisertib) [40] proceeding to phase III evaluation after demonstrating promising activity in a variety of tumour settings [41,42]. Interestingly, while numerous ATP-competitive and allosteric ligands for Aurora A have been developed, some of which can specifically target the highly similar Aurora A and Aurora B, no type IV (covalent) inhibitors of Aurora kinases have yet been reported.

Coenzyme A (CoA) is essential for the viability of all living cells where it is a major regulator of cellular metabolism [43,44]. The CoA biosynthetic pathway is conserved in prokaryotes and eukaryotes and requires five enzymatic steps, involving sequential conjugation of pantothenic acid (vitamin B5), cysteine and ATP [44]. The presence of a nucleotide moiety and a highly reactive thiol group permits broad diversity in biochemical reactions, which CoA employs to activate carbonyl-containing molecules and to generate covalent thioester derivatives such as Acetyl CoA, Malonyl CoA and 3-hydroxy-3-methylglutaryl CoA. Intracellular levels of CoA and its thioester-derivatives are tightly regulated by a variety of stimuli, including hormones, nutrients, metabolites and stresses [43,44]. The main rate-limiting enzyme in CoA biosynthesis is pantothenate kinase which initiates the biosynthetic pathway [45]. Furthermore, the activity of the last enzyme in the pathway, CoA synthase, is regulated by extracellular stimuli and stress response [[46], [47], [48], [49]].

CoA and its thioester derivatives are strategically positioned at the crossroads of cellular anabolic and catabolic pathways, controlling the Krebs cycle, ketogenesis, the biosynthesis of proteins, lipids and cholesterol, oxidation of fatty acids and degradation of amino acids. In addition, they are also involved in the regulation of gene expression and cellular metabolism via post-translational modifications, such as protein acetylation, butyrylation, malonylation and succinylation. Abnormal biosynthesis and homeostasis of CoA and its derivatives is associated with various human pathologies, including neurodegeneration, cancer, metabolic disorders and cardiac hypertrophy [44].

Protein CoAlation is a recently described post-translational modification (PTM) that involves covalent modification of cysteine residues by CoA [50]. New research tools and methodologies have revealed protein CoAlation as a widespread and reversible PTM involved in cellular redox regulation [51]. To date, more than one thousand proteins have been found to be CoAlated in a variety of prokaryotic and eukaryotic cells [50,52]. Protein CoAlation occurs at a low level in exponentialy growing cells, but is strongly induced in reponse to various oxidising agents, including H_2_O_2_, diamide, menadione and t-butyl hydroperoxide. Protein CoAlation alters the molecular mass, charge, and activity of modified proteins, and is thought to protect them from irreversible over-oxidation.

The presence of exposed cysteine residues on the surface of proteins provides a simple, sometimes reversible, mechanism for post-translational modification of cell signaling proteins in response to a wide variety of redox conditions. Most protein classes contain surface-exposed Cys residues, and for the protein kinase superfamily, this knowledge has been exploited to develop covalent (usually irreversible) ATP-dependent kinase inhibitors with clinical utility [53,54]. Interestingly, oxidative stress has marked effects on the cell cycle and proliferation [55], and redox regulation of signaling-active components such as tyrosine phosphatases [56] and CDC25 [57] contribute to redox-regulated cell cycle checkpoints. More recently, the effects of oxidative stress on abnormal spindle dynamics have been correlated with Aurora A inhibition [58]. However, whether CoA modifies and/or regulates protein kinases currently remains unknown.

In this study, we report Aurora A inhibition through a covalent interaction with CoA. We found that CoA is a specific ATP-competitive Aurora A inhibitor *in vitro*, and employed a combination of biochemical, structural, mass spectrometry and cellular studies to confirm a preferential mode of CoA binding to the active, phosphorylated, Aurora A conformation. Structural validation of the CoA binding mode confirms that it exploits Thr 217 in the ATP binding site, a locus that distinguishes Aurora A from Aurora B [31,40], to maintain a unique interaction with the 3′-phospho-ADP moiety of CoA, positioning it for formation of an unprecedented covalent bond between the pantetheine thiol and Cys 290, a remote, but highly conserved, Aurora A residue located in the activation loop. In support of this model, covalent modification of Aurora A by CoA was shown to be stimulated in human cells by oxidising agents, which also induced phosphorylation at Thr 288 and DTT-dependent dimerization of Aurora A. Furthermore, microinjection of CoA in mouse oocytes perturbed spindle formation and chromosome alignment *in vivo*. Taken together, our data reveal Aurora A as a novel target of the key metabolic regulator CoA, and opens the door for the development of a new class of small molecule inhibitor that exploits a ‘dual-anchor’ mechanism, bridging the Aurora A ATP-binding site and the activation segment.

## 2 Results

### 2.1 Kinase profiling screen with coenzyme A reveals specific inhibition of Aurora A

**Fig. 1:**
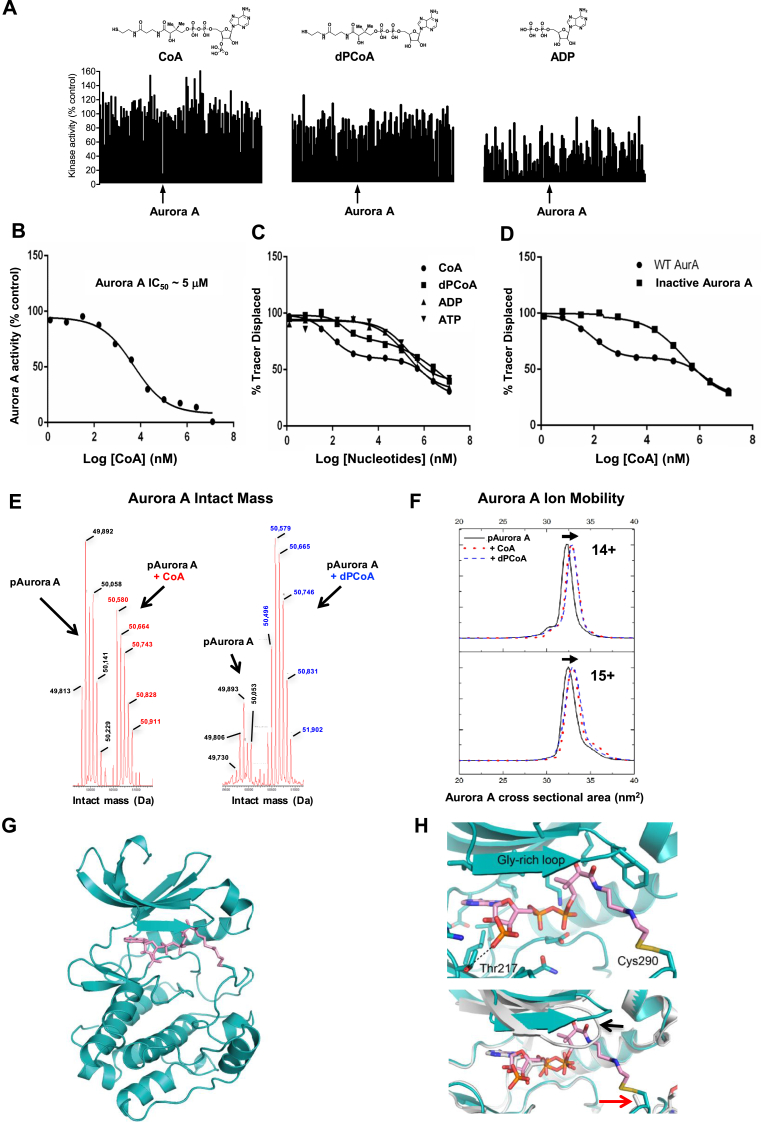
**Coenzyme A binds directly to Aurora A and inhibits catalytic activity.(A)** Kinase profiling screen reveals selective inhibition of Aurora A by CoA. The effect of CoA, dpCoA and ADP on the activity of 117 kinases was assayed using a radioactive filter-binding assay at the International Centre for Kinase Profiling, Dundee University. Each compound was tested at 100 μM final concentration in the presence of the indicated concentrations of ATP for each kinase (Table 1). Schematic structures of the tested compounds are shown above. **(B)** CoA inhibits Aurora A *in vitro*. Kinase activity of recombinant full-length His-Aurora A was assayed by measuring incorporation of γ^33^P-ATP into myelin basic protein in the presence of 5 μM ATP and an 11-point serial dilution of CoA. **(C)** Analysis of binding kinetics of CoA, dpCoA, ADP and ATP towards active (pT288-phosphorylated) Aurora A using a Lanthascreen Eu Kinase Binding assay **(D)** CoA preferentially binds to the active, pT288 phosphorylated form of Aurora A, when compared to the catalytically inactive dephosphorylated kinase. Dephosphorylation of His-Aurora A was carried out in the presence of recombinant PP1 phosphatase. **(E)** Intact mass analysis of phosphorylated Aurora A incubated with CoA in the absence of DTT. Covalent incubation of CoA with Aurora A generates a population of phosphorylated Aurora A (pAurora A) alongside covalent adducts containing an extra mass attributable to CoA (pAurora A + CoA). **(F)** Ion Mobility spectra and calculated cross sectional area (^TW^CCS_N2→He_) for [M+14H]^14+^ and [M+15H]^15+^ ions of pT288 Aurora A measured in the absence (black line) or presence of CoA (red lines) or dephospho-CoA (blue lines). Overlapping conformations of Aurora A are shown, the more extended of which have an increased mean cross sectional area associated with the presence of CoA and dephospho-CoA. **(G)** Crystal structure of Aurora A (teal) in complex with CoA (pink). **(H)** Upper panel, magnified view of CoA and its interactions with Aurora A, highlighting the side chains of Thr 217 and Cys 290, which forms a covalent bond with CoA. Lower panel, superposition of Aurora A/CoA with Aurora A/ADP (PDB code 1OL7), showing the shifted position of the Gly-rich loop (black arrow) and the equivalent position of Cys 290 (red arrow). Note that the side chain of Cys 290 was modelled without the sulfur atom in the Aurora A/ADP structure, due to weak electron density. (For interpretation of the references to color in this figure legend, the reader is referred to the Web version of this article.)

CoA and ATP share an ADP moiety, which we hypothesised might lead to a serendipitous modulation of the activity of protein kinases by CoA. We tested this notion in a human kinome-wide activity-based screen (MRC National Centre for Protein Kinase Profiling, University of Dundee). Profiling of 117 protein kinases with CoA, dephospho-CoA (dpCoA) and ADP provided clear evidence that CoA is a relatively selective inhibitor for human Aurora A (Fig. 1A and Table 1). Moreover, CoA inhibited Aurora A with marked selectivity (87% Aurora A inhibition at 100 μM, n = 3) when compared to ADP, a very non-specific kinase inhibitor that operates based on its close similarity to ATP. Only a few other kinases, including the tyrosine kinases SRC and YES, were inhibited ≤50% by CoA under these conditions. Interestingly, inhibition of Aurora B by CoA was also detected (46% inhibition at 100 μM compound), while Aurora C was not present in the screen (Table 1). dPCoA, which lacks the 3′-phosphorylated nucleotide, exhibited intermediate specificity between CoA and ADP, raising the possibility that both the pantetheine and 3′-phosphate moieties of CoA could be important for optimal inhibition of Aurora A (Fig. 1A). Further analysis confirmed that CoA inhibits active Thr 288 phosphorylated (recombinant) Aurora A with an IC_50_ of 5 μM in the presence of 5 μM ATP nucleotide *in vitro* (Fig. 1B). Using CoA Sepharose as an affinity matrix, we confirmed that endogenous Aurora A present in exponentially growing human HepG2 cells interacts specifically with conjugated CoA, when compared to beads alone (Supplementary Fig. S1). Furthermore, bound Aurora A was readily eluted from CoA Sepharose with an excess of CoA.

**Table 1:** **Kinase profiling of CoA, dpCoA and ADP.** The ability of CoA, dpCoA or ADP (100 μM final concentration of each compound and the indicated concentrations of ATP) to inhibit the phosphotransferase activity of 117 kinases was determined using a radioactive filter-binding assay at the International Centre for Kinase Profiling, DSTT, University of Dundee. Kinase activity in the absence of inhibitor is taken as 100%.

Kinase	CoA	ADP	dpCoA	ATP in assay (μM)
ABL	111.2	22.9	98.0	5
AMPK	90.8	54.1	75.9	50
ASK1	129.1	70.2	88.6	50
Aurora A	13.4	38.0	29.9	5
Aurora B	54.3	47.8	55.1	20
BRK	114.1	41.3	83.1	20
BRSK1	110.4	42.8	96.4	20
BRSK2	114.7	52.4	88.8	50
BTK	67.9	18.2	30.6	50
CAMK1	123.6	80.1	125.6	50
CAMKK	97.4	52.9	71.4	20
CDK2-Cyclin A	93.2	26.3	81.1	20
CHK1	122.6	47.6	78.8	20
CHK2	88.9	43.2	56.0	20
CK1δ	80.8	57.6	80.4	20
CK2α	80.1	5.4	39.8	5
CLK2	108.9	33.9	80.1	5
CSK	92.6	18.5	54.9	20
DAPK1	106.2	10.3	64.4	5
DYRK1A	93.1	75.9	90.2	50
DYRK2	102.4	42.4	77.0	50
DYRK3	95.1	16.2	91.2	5
EF2K	67.1	86.2	103.0	5
EPH-A2	102.0	45.0	99.2	50
EPH-A4	114.6	32.5	75.6	50
EPH-B1	116.9	11.6	80.0	20
EPH-B2	117.3	14.3	91.1	20
EPH-B3	104.2	18.0	94.4	20
EPH-B4	106.8	9.8	62.1	50
ERK1	118.2	67.7	114.0	5
ERK2	119.7	54.4	81.7	20
ERK8	101.4	16.6	38.5	5
FGF-R1	60.0	41.7	31.4	20
GCK	108.8	56.5	71.2	20
GSK3β	94.7	18.2	72.1	5
HER4	100.5	29.1	75.7	5
HIPK1	115.3	31.7	92.8	20
HIPK2	153.3	16.5	73.4	5
HIPK3	123.1	34.2	91.5	20
IKKβ	90.5	26.6	48.0	5
IKKε	125.2	73.8	91.4	50
IRAK4	89.1	82.1	74.8	20
JAK2	105.5	7.1	62.5	5
JNK1	85.4	53.9	82.4	20
JNK2	93.9	57.3	76.1	20
JNK3	101.3	58.6	63.2	20
Lck	100.4	35.1	69.0	50
LKB1	103.3	71.0	81.1	20
MAPKAP-K2	93.1	53.0	81.7	20
MAPKAP-K3	98.4	25.7	63.0	20
MARK1	95.3	28.1	68.0	20
MARK2	93.1	25.0	81.5	20
MARK3	88.9	14.8	46.2	5
MARK4	87.7	49.1	86.6	50
MEKK1	96.3	68.1	105.8	20
MELK	76.3	63.5	104.0	50
MINK1	106.3	49.4	103.9	50
MKK1	107.2	16.4	52.3	5
MKK2	103.0	35.4	53.0	5
MKK6	88.6	39.1	88.2	50
MLK1	109.9	41.4	76.0	20
MLK3	70.2	46.8	61.9	20
MNK1	150.3	60.7	84.2	50
MNK2	137.5	41.2	87.1	50
MSPK1	101.8	94.1	101.7	50
MSK1	106.4	53.3	70.5	20
MST2	118.8	40.5	72.5	20
MST4	107.0	56.9	89.7	20
NEK2α	148.5	42.1	100.4	50
NEK6	118.4	26.6	69.6	50
NUAK1	98.3	32.4	68.3	20
p38α MAPK	112.5	60.3	87.7	50
p38β MAPK	159.6	51.1	94.5	20
p38δ MAPK	128.0	37.3	71.3	5
p38**γ** MAPK	127.2	26.0	65.9	5
PAK2	88.8	90.3	111.9	20
PAK4	69.9	73.8	75.9	5
PAK5	70.6	79.5	86.0	20
PAK6	98.4	78.4	100.7	20
PDK1	113.3	23.3	91.2	20
PHKγ1	105.1	75.6	90.9	50
PIM1	103.0	46.1	97.7	20
PIM2	111.0	9.8	62.9	5
PIM3	121.7	36.1	96.7	20
PKA	98.9	27.8	81.3	20
PKCα	104.2	38.6	76.8	5
PKCβ	118.3	79.2	105.1	50
PKCα	78.1	76.5	88.9	20
PKCζ	99.3	37.7	89.9	5
PKCγ	70.2	71.9	66.4	20
PKD1	111.9	56.2	104.3	50
PLK1	101.3	52.2	100.0	5
PRAK	99.7	15.2	77.7	20
PRK2	71.3	20.8	67.5	5
RIPK2	131.4	46.0	88.4	20
ROCK 2	94.3	57.8	96.4	20
RSK1	97.2	58.5	98.9	50
RSK2	96.5	47.9	62.9	50
S6K1	89.1	35.5	30.5	20
SGK1	102.8	38.7	74.3	20
SmMLCK	92.1	73.0	91.3	20
Src	43.8	31.8	8.9	50
SRPK1	116.3	60.9	89.1	50
STK33	71.8	54.6	83.3	50
SYK	98.3	70.9	105.9	20
TAK1	115.3	15.8	79.6	5
TAO1	113.2	34.0	98.3	20
TBK1	106.3	95.1	109.6	50
TIE2	93.0	33.1	83.4	20
TTK	101.3	54.7	75.8	20
YES1	49.0	18.6	11.5	20
ZAP70	89.7	13.2	79.8	5
IGF-1R	93.0	–	–	5
IR	100.0	–	–	20
IRR	92.0	–	–	5
TrkA	104.0	–	–	20
VEGFR	81.0	–	–	20

Next, we quantified the binding kinetics of CoA, dpCoA, ADP and ATP for catalytically active (Thr 288 phosphorylated) and catalytically inactive (non-phosphorylated) forms of Aurora A. A Lanthascreen Eu Kinase FRET Binding Assay was employed to evaluate the interaction of CoA with Aurora A, and tease apart any molecular contributions made by the pantetheine tail and nucleotide 3′-phosphate. We found that the displacement curves for CoA and dpCoA were biphasic, indicating the presence of two potential binding sites with higher and lower affinities towards CoA (Fig. 1C). Bacterially expressed Aurora A proteins auto-phosphorylate, resulting in a heterogeneous mixture containing active and inactive Aurora A as a result of sub-stoichiometric phosphorylation of different sites [14]. Importantly, treatment of bacterially expressed Aurora A with protein phosphatase 1, which results in Thr 288 dephosphorylation [9], removed the high affinity-binding site, confirming that this was related to the activation (phosphorylation) state of the kinase (Fig. 1D). We next determined the affinity of interaction by titrating Aurora A with CoA, dpCoA and ADP, and compared them with values obtained from *in vitro* kinase (phosphotransferase) assays (Table 2). Under the assay conditions used, the apparent IC_50_ for the interaction of active Aurora A with CoA and dPCoA were 82 nM and 294 nM, respectively, whilst the IC_50_ value for ADP was approximately 177 μM. Taken together, these results clearly demonstrate that CoA and dpCoA preferentially bind to the active (Thr 288-phosphorylated) form of Aurora A, and suggest an involvement of the pantetheine tail and the 3′-phosphate of CoA in mediating a potentially specific interaction with Aurora A.

**Table 2:** **CoA preferentially binds to active, T288 phosphorylated Aurora A**. The binding of T288 phosphorylated Aurora A (‘active’), or unphosphorylated Aurora A (‘inactive’), was assessed for each compound and the inhibitory constants were calculated. The data confirm that both the pantetheine and the 3′-phosphate of CoA participate in the interaction between Aurora A and CoA.

Compound	Aurora A (active) IC_50_ (μM), binding assay	Aurora A (inactive) IC_50_ (μM), binding assay	Aurora A (active) IC_50_ (μM), activity assay
ATP	0.2	3.0	–
ADP	0.17	1.3	54
dpCoA	0.0003	>100	17
CoA	0.00001	0.28	5

### 2.2 Biophysical and structural analysis of the Aurora A:CoA complex

To confirm the interaction between CoA and Aurora A, we pre-incubated phosphorylated Aurora A (containing pThr 288) with a molar excess of CoA or dPCoA and analysed the complexes using intact mass spectrometry (MS). Upon incubation, a sub-stoichiometric shift was observed for each of the differentially phosphorylated intact Aurora A species present in the preparation, consistent with the formation of phosphorylated Aurora A adducts, each containing a single molecule of CoA or dPCoA (Fig. 1E). We also evaluated effects of CoA and dPCoA on the gas-phase conformation of Aurora A under native MS conditions using Ion Mobility Mass Spectrometry (IM-MS), which is a recently-developed approach that can be used to probe kinase conformational dynamics [69]. The calculated cross-sectional (CCS) area of intact Aurora A (nm [2]) in both 14^+^ and 15^+^ charge states increased after pre-incubation with both CoA or dPCoA, consistent with their ability to bind to Aurora A and induce a conformational change. The increase in Aurora A CCS upon binding of either CoA or dPCoA, suggests that it is the covalent attachment of the CoA pantetheine tail that drives the conformational change in Aurora A (Fig. 1F).

To gain further insight into the binding mode of CoA for active Aurora A, we next determined the crystal structure of the complex to 2.5 Å resolution (Table 3, Fig. 1G). As predicted, CoA occupies the canonical ATP binding site of Aurora A, between the N-lobe and C-lobe of the kinase. Remarkably, the extended CoA pantetheine moiety stretches away from the ATP site towards the kinase activation loop, forming a disulfide bond with the side chain of Cys 290 adjacent to Thr 288, the phosphorylated residue (Fig. 1H). Unexpectedly, the pantothenic acid moiety of CoA interacts with the tip of the Gly-rich loop (aa142-145) and displaces it from the position found in Aurora A/ADP complexes, consistent with our IM-MS data. Interestingly, the 3′ phosphate group of CoA is also ideally positioned to form an H-bond with the side chain of Thr 217, which is evolutionary conserved in vertebrate Aurora A, but not Aurora B [31,40]. Our crystal structure of Aurora A/CoA was consistent with independent molecular modelling of Aurora A bound to CoA, based on a previously determined structure of Aurora A/ADP (Supplementary Fig. S2). The structure employed for modelling (PDB code 1OL7), was chosen based on previous knowledge that the side chain of Cys 290 is in a potentially suitable position to react chemically with the pantetheine tail of CoA. Importantly, most features of the experimental crystal structure were predicted by this model, including the specific interaction of the 3’-phosphate group of CoA with the side chain of Thr 217. One exception is the experimentally up-shifted position of the Gly-rich loop in the presence of CoA, which was unexpected based on the model, in which the Gly-rich loop resides in a canonical position, packed against the ADP phosphates.

**Table 3:** **Data collection and refinement statistics**.

	Aurora A 122–403/CoA	
**Data collection**		
Space group	*P*6_1_22	
Cell dimensions		
*a*, *b*, *c* (Å)	80.89, 80.89, 173.34	
α, β, γ (°)	90.00, 90.00, 120.00	
Resolution range (Å)	44.57–2.50 (2.54–2.50)^a^	
*R*_merge_ (%)	8.58 (171)	
*I/σI*	21.92 (1.61)	
Completeness (%)	100 (100)	
Redundancy	17.59 (15.07)	
**Refinement**		
Resolution (Å)	44.57–2.50	
No. reflections	12263	
*R*_work_/*R*_free_	20.44/25.21	
No. atoms		
Protein	2102	
Hetero	73	
Water	26	
Mean *B*-factors		
Protein	69.90	
Hetero	77.39	
Water Wilson *B*-factor	69.30 70.56	
r.m.s. deviations		
bond lengths (Å)	0.013	
bond angles (°) **MolProbity analysis** All-atom clash-score	1.23 9.17	
Rotamers outliers (%)	0	
Ramachandran outliers (%)	0	
Ramachandran favoured (%)	95.06	
MolProbity score	1.83	

a Values in parentheses are for highest-resolution shell.

### 2.3 Aurora A is CoAlated on Cys 290 *in vitro*

**Fig. 2:**
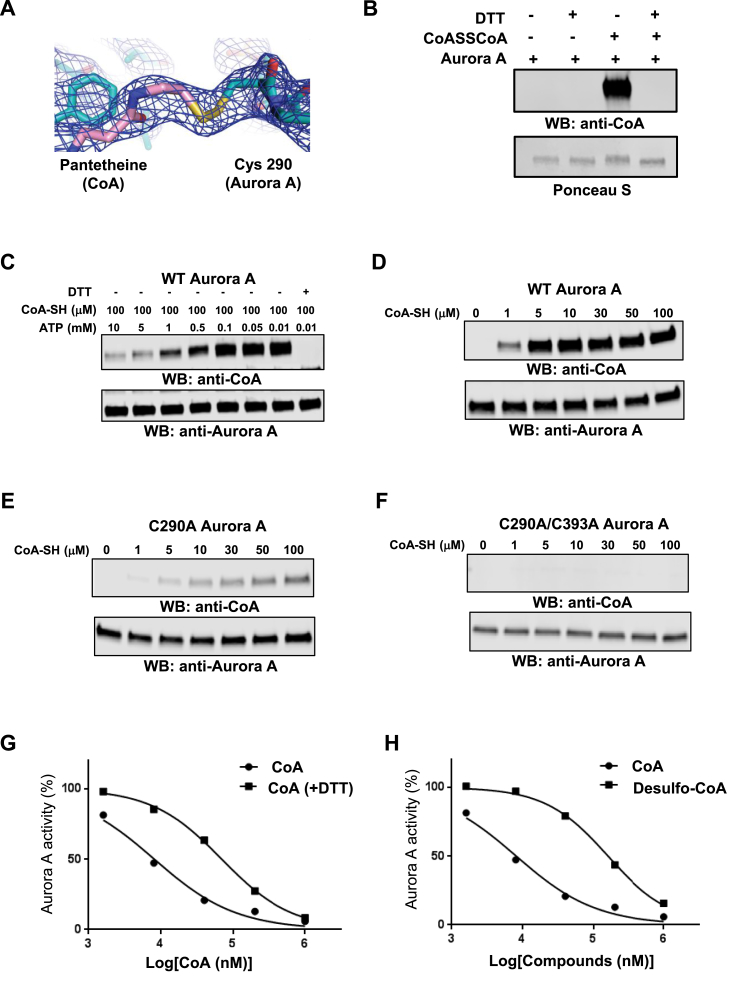
**CoA reversibly binds to Aurora A in an ATP-competitive manner through Cys 290. (A)** Electron density around the pantotheine tail region of CoA and Cys 290 of Aurora A, 2Fo-Fc map contoured at 1 σ. **(B)** Aurora A is covalently modified by CoA in a DTT-sensitive manner. *In vitro* CoAlated Aurora A was separated by SDS-PAGE in the presence or absence of DTT and immunoblotted with anti-CoA antibody. The membrane was stained with Ponceau red to visualize total Aurora A. **(C)** Binding of CoA to the ATP-binding pocket is required to facilitate covalent modification of Aurora A by CoA. *In vitro* CoAlation of Aurora A was carried out in the presence of 100 μM CoA and the indicated concentration of ATP. Generated samples were separated by SDS-PAGE in the presence or absence of DTT and immunoblotted with anti-CoA and anti-Aurora A antibodies. **(D)** WT Aurora A is efficiently CoAlated by CoA *in vitro*. **(E)** The C290A Aurora A mutant exhibits significantly reduced binding of CoA when compared to WT Aurora A. **(F)** The C290/393A Aurora A mutant is not covalently modified by CoA *in vitro*. *In vitro* CoAlation of WT Aurora A, C290A and C290/393A mutants was performed with the indicated concentration of CoA. The reaction mixtures were separated by SDS-PAGE and immunoblotted with anti-CoA and Aurora A antibodies. **(G)** The inhibitory effect of CoA towards Aurora A is reduced by DTT. Recombinant His-Aurora A was used to determine the IC_50_ value for CoA in the presence or absence of 1 mM DTT. **(H)** Reduced inhibitory effect of desulfo-CoA compared to CoA. Desulfo-CoA lacks the reactive SH group at the end of the pantetheine tail. (For interpretation of the references to color in this figure legend, the reader is referred to the Web version of this article.)

The electron density map unambiguously supports the formation of a covalent bond between the sulfur atoms in the side chain of Cys 290 and pantetheine thiol of CoA (Fig. 2A). To investigate the contribution of this bond to the interaction, we employed a validated *in vitro* CoAlation assay [50]. Here, recombinant Aurora A was pre-incubated with CoA, and the reaction mixture separated by SDS-PAGE under non-reducing conditions followed by immunoblotting with a monoclonal anti-CoA antibody. As shown in Fig. 2B, CoA interacts with Aurora A in a DTT-sensitive manner, suggesting a thiol-dependent covalent mode of binding. Ponceau staining of immunoblotted samples revealed that the mobility of Aurora A was retarded when CoA disulfide was present in the reaction mixtures. The addition of DTT completely abolished the observed electrophoretic mobility shift, indicating the formation of CoA–Aurora A mixed disulfides. LC-MS/MS analysis was employed to map CoA-modified cysteine(s) of *in vitro* CoAlated Aurora A. This analysis revealed that Cys290 is CoAlated in the RTpTLC^290^GTLDYPPEMIEGR peptide (Supplementary Fig. S3A), confirming the co-existence of Thr 288-phosphorylated (active) Aurora A and Cys 290-CoA *in vitro*. We also examined whether binding of the 3′-phosphate ADP moiety of CoA to the Aurora A ATP binding pocket primes the pantetheine thiol for disulfide bond formation with Cys 290 in the activation loop. *In vitro* CoAlation of Aurora A was carried out with 100 μM CoA and increasing concentrations of ATP (0–10 mM). Aurora A CoAlation gradually decreased in the presence of increasing concentrations of ATP, and was completely abolished by inclusion of 1 mM DTT (Fig. 2C). These observations suggest that occupancy of the ATP binding pocket by the 3′-phosphate ADP moiety of CoA is a pre-requisite for covalent modification of Aurora A (see below).

Aurora A has two surface-exposed cysteine residues present in the kinase domain: Cys 290 in the activation loop and Cys 393 in the C-terminal regulatory region [65]. Mutational analysis allowed us to demonstrate that covalent modification of the Cys290Ala mutant by CoA was dramatically reduced (by >95%) in comparison to wild-type Aurora A, while no binding was detected with the double Cys290/393Ala mutant (Fig. 2D–F). The dose-dependency of inhibition of active Aurora A by CoA was also examined in the absence and presence of a reducing agent (Fig. 2G). Aurora A was inhibited by CoA under both experimental conditions. However, the IC_50_ value (CoA) was an order of magnitude higher in the presence of DTT (47 μM), when compared to that obtained in the absence of DTT (5 μM). To explore the mode of regulation further, we tested the inhibitory effect of desulfo-CoA on Aurora A kinase activity. In this analogue of CoA, the reactive SH group at the end of the pantetheine tail is absent and unable to covalently modify Aurora A. Consistently, the dose response inhibition curve for desulfo-CoA was also shifted, resulting in a significantly higher IC_50_ value for Aurora A (160 μM), confirming the involvement of the CoA thiol group in the specific inhibition of Aurora A kinase activity (Fig. 2H).

### 2.4 The role of Thr 217 in mediating a selective interaction between Aurora A and CoA

**Fig. 3:**
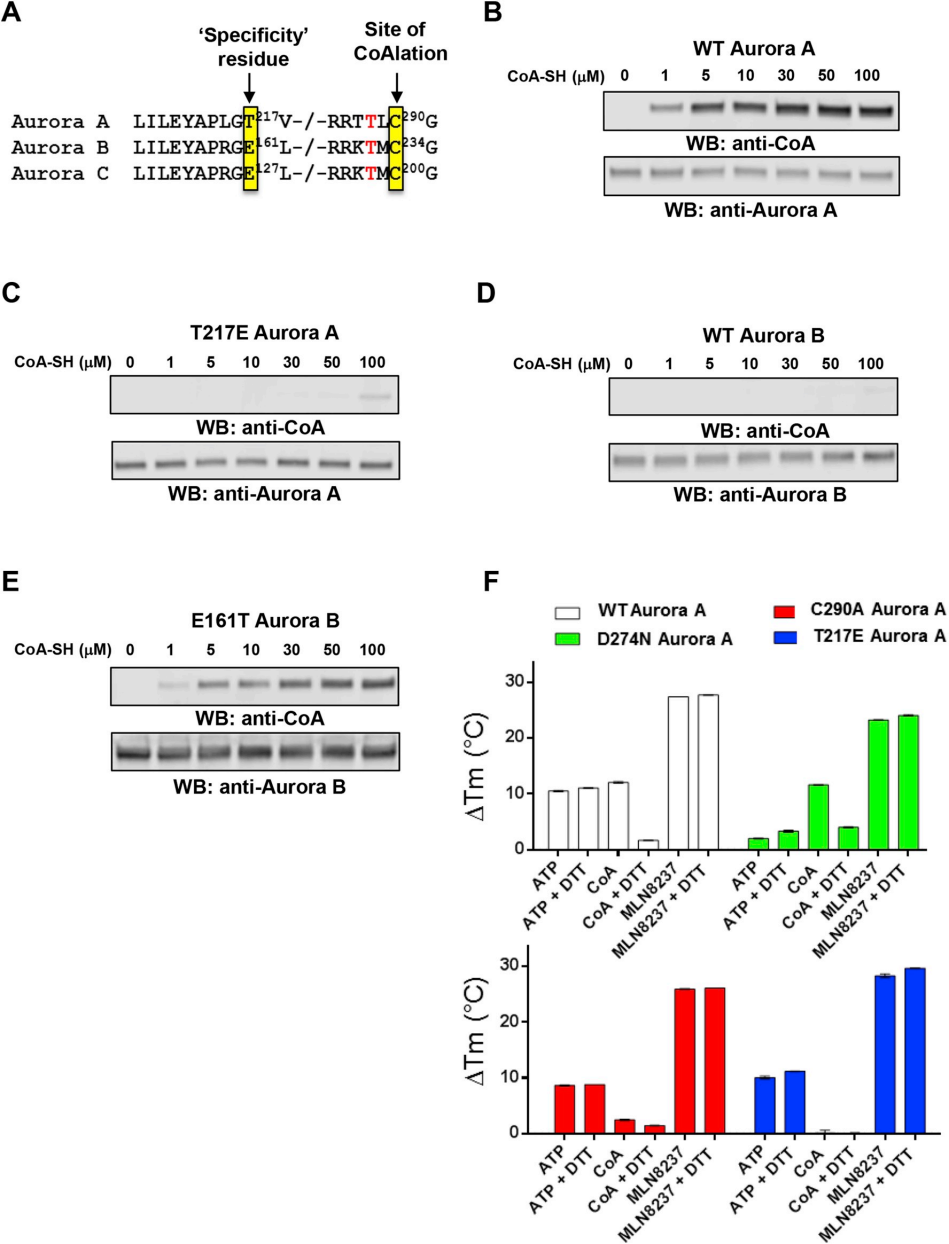
**Specificity of Aurora A interaction with CoA is controlled by Thr 217.(A)** Amino acid conservation in vertebrate Aurora kinases. Thr 217 defines Aurora A, and is changed to a Glu residue in Aurora B and C. Cys 290 (boxed) is invariant in all Aurora kinases, and lies in the activation segment adjacent to the phosphorylated Thr 288 (human Aurora A numbering). **(B)***In vitro* CoAlation of Aurora A. **(C)***In vitro* CoAlation of Aurora A is abolished in the Thr217Glu mutant. **(D)***In vitro* CoAlation is not detected with Aurora B. **(E)** The Glu161Thr Aurora B mutant is efficiently CoAlated. Experiments were performed with the indicated concentration of CoA. Reaction mixtures were separated by SDS-PAGE and immunoblotted with anti-CoA, Aurora A or Aurora B antibodies. **(F)** A thermal shift assay was employed to evaluate Aurora A binding to 5 mM ATP, 5 mM CoA or 0.1 mM MLN8237, in the presence and absence of 1 mM DTT, as indicated. Assays were performed with T 288 phosphorylated, active, Aurora A (open bars), kinase-dead, dephosphorylated Aurora A (Asp274Asn, green bars), Cys290Ala Aurora A (red bars) or Thr217Glu Aurora A (blue bars). Mean ΔT_m_ values ± SD (n = 3) were calculated by subtracting the control T_m_ value (buffer, no addition) from the measured T_m_ value. (For interpretation of the references to color in this figure legend, the reader is referred to the Web version of this article.)

We next sought to identify the basis of selectivity of CoA for Aurora A over other protein kinases. To do this, we focussed on the closely related Aurora A and B kinases, which share 70% sequence identity in the kinase domain (Fig. 3A). The putative hydrogen bond between the –OH side chain of Thr 217 in Aurora A and the 3′-phosphate of the ribose ring of CoA (Fig. 1H) was of particular interest since Thr 217 is known to contribute to the specificity of MLN8054, a selective Aurora A inhibitor [40], whereas the corresponding residue Glu 177 in Aurora B prevents compound binding, likely due to electrostatic repulsion [31,40]. We compared covalent modification of Aurora A and Aurora B using an *in vitro* CoAlation assay (Fig. 3B, D). This analysis revealed strong CoAlation of Aurora A, while Aurora B was CoAlated at an extremely low level under optimal Aurora A assay conditions. Mutational analysis showed that the Thr217Glu mutant of Aurora A exhibits significantly reduced CoA binding compared to the wild-type kinase (Fig. 3C). In contrast, a reciprocal Glu177Thr Aurora B mutant protein exhibited strong binding to CoA (Fig. 3E). Together, these data indicate that both the 3′-phosphate ADP moiety and the thiol group of CoA are involved in mediating specificity-determining interactions with Aurora A *via* targeting of Thr 217 and Cys 290 respectively.

### 2.5 Thermal shift-based detection of the Aurora A/CoA interaction

To probe thermal effects of CoA in the presence and absence of DTT, we profiled Aurora A ligand binding in solution (Fig. 3F). WT Aurora A is phosphorylated and catalytically active, whereas D274 N Aurora A cannot bind to ATP and therefore remains dephosphorylated after isolation from bacteria [7]. In contrast, C290A Aurora A is catalytically active, but lacks the thiol-containing Cys residue in the kinase activation segment (Fig. 3A), while T217E Aurora A mimics Aurora B by swopping a polar Thr for a charged Glu (see above). Thermal melting profiles [60] for each protein were obtained in the presence and absence of DTT, ATP, CoA or the kinase inhibitor MLN8237. Binding of ATP, CoA and MLN8237 induced a marked change in thermal stability of WT Aurora A, which was dependent on non-reducing conditions in the case of CoA, and entirely consistent with our findings employing Aurora A isolated from human cells (Fig. 2B). Interestingly, CoA was still able to interact with kinase-dead D274 N (inactive) Aurora A in a DTT-dependent manner, although the interaction of this mutant with Mg-ATP was severely blunted, as expected (green bars). In contrast, C290A Aurora A still bound to ATP, but was deficient in CoA-induced thermal stabilization (red bars). Finally, a CoA-induced thermal shift was completely abolished in the T217E Aurora A mutant, in contrast to stabilization by either ATP or MLN8237, confirming the importance of a Thr in Aurora A for CoA binding.

### 2.6 Binding of TPX2 protects Aurora A from covalent modification and inhibition by CoA

**Fig. 4:**
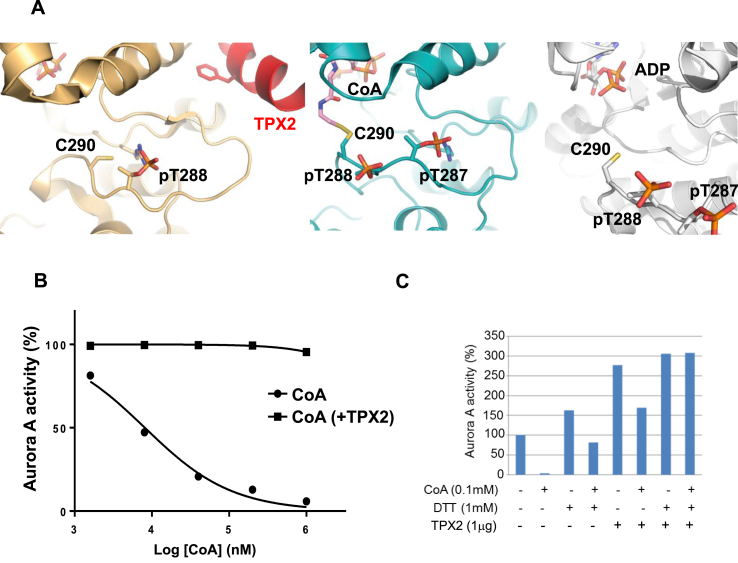
**Inhibitory effect of TPX2 binding on CoA interaction with Aurora A and position of pT288.(A)** Magnified views of the activation loop of Thr 288-phosphorylated Aurora A in the crystal structures of complexes with: ADP/TPX2, PDB code 1OL5; CoA (center); or ADP, PDB code 1OL7 (right). Note that in the ADP complex, the sulfur on the side chain of Cys 290 is not included in the experimental model but is shown here, and the β3-αC loop removed for clarity. **(B)** Aurora A is resistant to inhibition by CoA in the presence of TPX2 1–43 peptide. Kinase activity of recombinant His-Aurora A was assayed by measuring incorporation of γ^33^P-ATP into myelin basic protein in the presence or absence of N-terminal fragment of TPX2 (residues 1–43) and serial dilutions of CoA **(C)** Pre-incubation of Aurora A with CoA inhibits TPX2-induced kinase activation in a DTT-sensitive manner.Recombinant His-Aurora A was preincubated with 100 μM CoA before the *in vitro* kinase assay which was performed in the presence or absence of the N-terminal fragment of TPX2 (residues 1–43) and DTT.

In vertebrates, TPX2 enhances Aurora A autophosphorylation and protects it from dephosphorylation by phosphatases. Biochemical and crystallographic analyses have revealed the molecular mechanism by which the N-terminal fragment of TPX2 (residues 1–43) interacts with Aurora A, and locks it in a stable active conformation, also protecting it from dephosphorylation by PP1 phosphatase (Fig. 4A, left). Protection from enzymatic dephosphorylation [9] occurs because the phosphate group of Thr 288 is buried in the TPX2-bound form [10]. Interestingly, the side chain of Cys 290 is also buried in the TPX2-bound, active conformation of Aurora A. Furthermore, the thiol group points away from the ATP binding side, whereas it is exposed in structures of active Aurora A obtained in the absence of TPX2 in which CoA (Fig. 4A, middle) or ADP (Fig. 4A, right) are bound. Based on this observation, we next investigated whether the inhibitory effect of CoA on Aurora A was modulated by TPX2. Dose inhibition curves of Aurora A by CoA were generated in the presence and absence of TPX2. As shown in Fig. 4B, the presence of the TPX2 1–43 fragment completely abrogated the inhibitory effect of CoA on Aurora A. Moreover, pre-incubation of Aurora A with CoA attenuated TPX2-induced kinase activation in a DTT-sensitive manner (Fig. 4C). These findings indicate that binding of TPX2 protects Aurora A not only from dephosphorylation of Thr 288 by phosphatases [9], but that it also prevents covalent modification (and therefore inhibition) by CoA.

### 2.7 Oxidative stress induces Aurora A CoAlation in cells

**Fig. 5:**
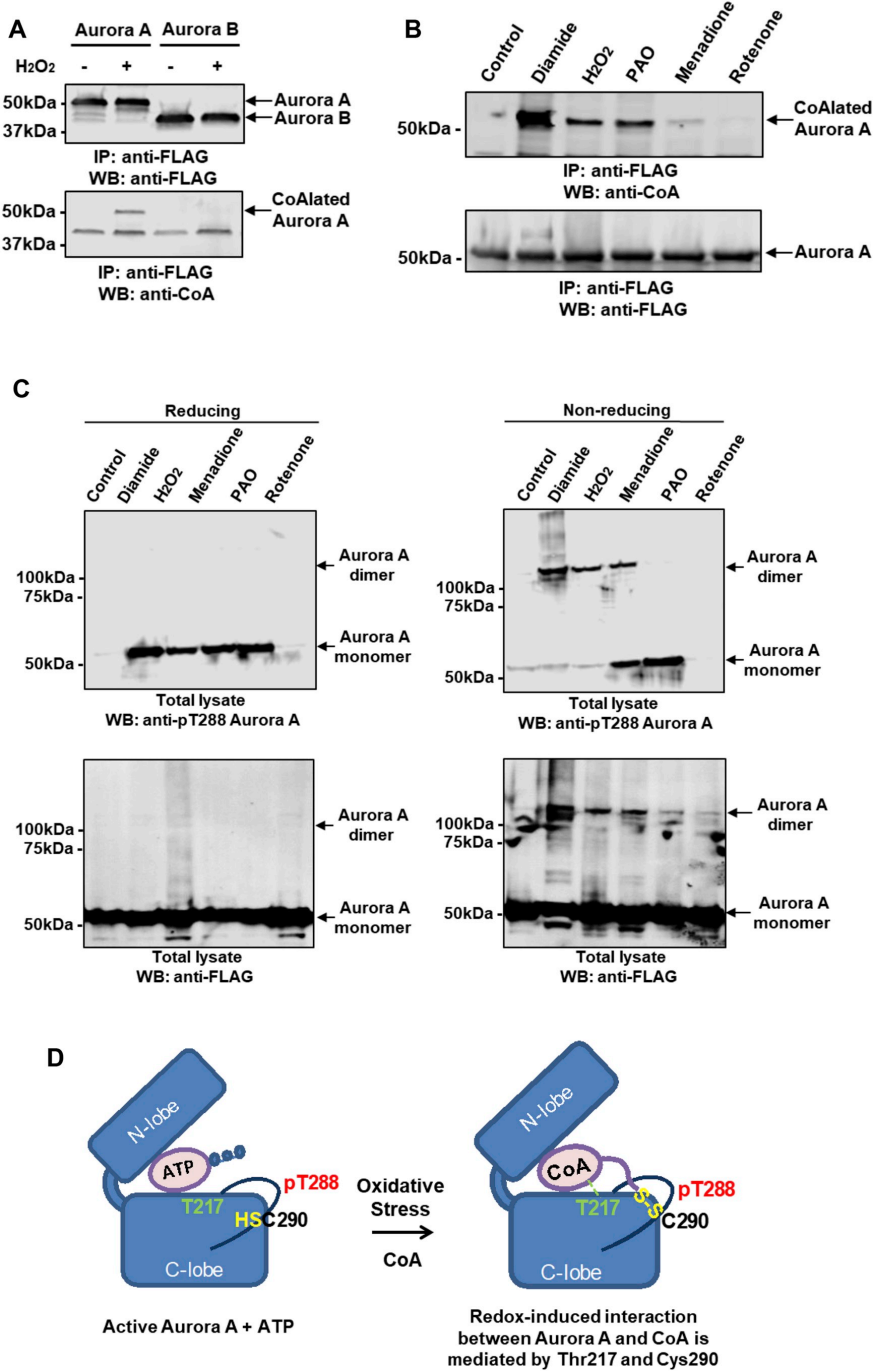
**Oxidative stress induces Aurora A CoAlation in human cells.(A)** Aurora A CoAlation is induced in cellular response to H_2_O_2_. FLAG-tagged WT Aurora A and WT Aurora B were transiently overexpressed in HEK293/Pank1β. Transfected cells were treated for 30 min with 0.25 mM  H_2_O_2_. Overexpressed proteins were immunoprecipitated with an anti-FLAG antibody and the immune complexes immunoblotted with anti-CoA and anti-FLAG antibodies. **(B)** Oxidising agents promote Aurora A CoAlation *in vivo*. FLAG-tagged WT Aurora A was transiently over expressed in HEK293/Pank1β. Transfected cells were treated for 30 min with a panel of oxidising agents (250 μM H_2_O_2_, 500 μM diamide, 50 μM menadione, 10 μM phenylarsine oxide and 1 μM rotenone). Transiently expressed proteins were immunoprecipitated with an anti-FLAG antibody, separated by SDS-PAGE under non-reducing conditions and immunoblotted with anti-CoA antibodies. **(C)** Phosphorylation at Thr 288 and dimerization of Aurora A are induced in cells by oxidising agents. FLAG-tagged WT Aurora A was transiently overexpressed in HEK293/Pank1β. Transfected cells were treated for 30 min with a panel of oxidising agents (250 μM H_2_O_2_, 500 μM diamide, 50 μM menadione, 10 μM phenylarsine oxide and 1 μM rotenone). Transiently expressed proteins were immunoprecipitated with an anti-FLAG antibody, separated by SDS-PAGE under reducing and non-reducing conditions and immunoblotted with anti-FLAG and anti-pT288 Aurora A antibodies. **(D)** Schematic illustration showing the key features of the ‘dual anchor’ mechanism for interaction of CoA with Thr 217 and Cys 290 in Aurora A.

Extensive protein CoAlation is observed in mammalian cells and tissues exposed to oxidative and metabolic stress [50]. We therefore examined whether covalent modification of Aurora A by CoA is induced in cells after exposure to various oxidative stresses. We exploited HEK293/Pank1β cells, which stably overexpress pantothenate kinase 1 β (Pank1β), the major rate-limiting enzyme in CoA biosynthesis [50]. It has been shown previously that Pank1β overexpression induces a significant increase in CoA biosynthesis [45]. These cells are also ideal for evaluating protein CoAlation during the cellular response to oxidative and metabolic stress [50]. Accordingly, cells were transiently transfected with plasmids encoding N-terminally FLAG-tagged Aurora A and Aurora B, and then treated with 500 μM  H_2_O_2_ prior to lysis. Immunoprecipitation of transiently expressed FLAG-tagged proteins followed by immunoblotting with anti-CoA antibody revealed specific CoAlation of Aurora A in cells after exposure to H_2_O_2_, whereas Aurora B was poorly CoAlated (Fig. 5A). To map the site(s) of Aurora A CoAlation, transiently overexpressed FLAG-Aurora A was immunoprecipitated from cells exposed to H_2_O_2_, processed and analysed by LC-MS/MS. This analysis revealed a CoAlated Aurora A peptide derived from the activation segment with the sequence TTLC^290^GTLDYLPPEMIRGR, confirming covalent modification in cells (Supplementary Fig. S3B), and consistent with CoAlation of purified Aurora A *in vitro* (Fig. 2B). Next, Aurora A CoAlation was examined in cells exposed to a diverse range of oxidising agents. As shown in Fig. 5B, CoAlation of transiently expressed Aurora A was strongly induced in cells treated with diamide and to a lesser extent after exposure to hydrogen peroxide, menadione or phenylarsine oxide (PAO). It was recently reported that ROS-induced cell cycle arrest promotes hyperphosphorylation of Aurora A leading to abnormal mitotic spindle assembly and significant mitotic delay [58]. Therefore, we examined phosphorylation of Aurora A at Thr 288 by probing the same samples with an anti-pT288 antibody. We confirmed that treatment of cells with diamide, H_2_O_2,_ menadione and PAO promoted an increase in Aurora A phosphorylation at Thr 288 when samples were separated in the presence of the reducing agent DTT (Fig. 5C). However, when the samples were separated under non-reducing conditions and probed with anti-pT288 and anti-FLAG antibodies, the formation of Aurora A dimers was clearly observed in cells treated with diamide, H_2_O_2_ and menadione (Fig. 5C). In support of these findings, we found that brief exposure of purified Aurora A to peroxide increased the appearance of a putative Aurora A dimer, which was prevented by mutating Cys 290 to Ala. The dimer was sensitive to DTT exposure, based on a comparative reducing and non-reducing SDS-PAGE analysis (Supplementary Fig. S4). Taken together, these results allow us to propose a simple model for regulation of Aurora A by oxidative stress in cells (Fig. 5D). In exponentially growing cells, Aurora A carries out many of its cellular functions by using ATP as substrate for protein phosphorylation. The binding of CoA to the Aurora A ATP binding pocket *via* the ADP moiety is effectively outcompeted by ATP (Fig. 5D, left panel). In contrast, exposure of cells to oxidative stress induces covalent binding of CoA to phosphorylated Aurora A (Fig. 5D, right panel), which is accompanied by a loss of catalytic activity and altered cell signaling.

### 2.8 CoA microinjection studies in mouse oocytes

**Fig. 6:**
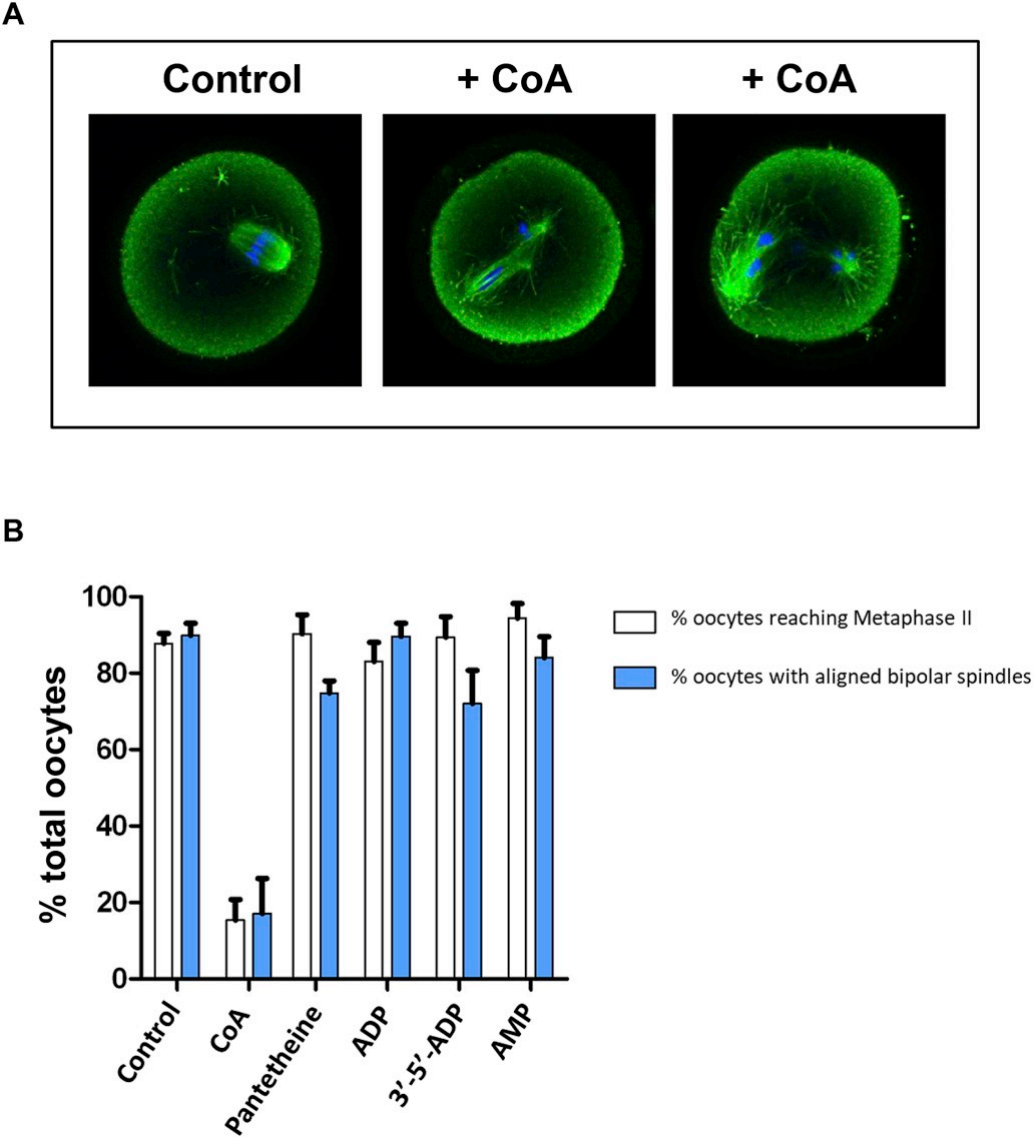
**Microinjection of CoA causes abnormal spindles and chromosome misalignment in mouse oocytes.** **(A)** Mouse oocytes arrested at the GV stage were injected with CoA or related compounds, representing different determinants of the CoA molecule. 17 hrs after GV release, cells were fixed and stained for alpha-tubulin (green) or DNA (blue). Representative examples of control and CoA-injected oocytes are shown. **(B)** The numbers of mouse oocytes with normal and abnormal spindles were recorded. Data shown are from n = 2 experiments. As a control, ~90% of control water-injected oocytes were in possession of a normal metaphase II bipolar spindle with aligned chromosomes. (For interpretation of the references to color in this figure legend, the reader is referred to the Web version of this article.)

To study the cell cycle effects of increased CoA *in vivo*, we employed two established microinjection models. Catalytically-active Aurora A is essential for normal spindle formation during mouse oocyte maturation [16,71], and blocks mitotic cell cycles after *Xenopus* embryo microinjection [72,73]. Mouse oocytes arrested at the GV stage were injected with different concentrations of CoA, and 17 h after GV release, oocytes were fixed and stained for α-tubulin (green) and DNA (blue) to observe effects on spindle formation, which are under the control of Aurora A. We found that microinjection of CoA markedly increased the number of oocytes with atypical spindles and misaligned chromosomes in a dose-dependent manner (Fig. 6A). Our *in vitro* studies convincingly demonstrated the importance of both the pantetheine tail and the 3’-phosphate of CoA in mediating a high-affinity interaction with Aurora A (Fig. 1). We therefore examined the effect of injected CoA, or related compounds lacking different regions of the CoA molecule, to cause abnormal spindle formation and misalignment of chromosomes in mouse oocytes. We found that CoA, but not pantetheine, ADP or AMP, disrupted both bipolar spindle formation and chromosome alignment *in vivo* (Fig. 6B). These data confirm that the pantetheine tail is required for inhibitory spindle effects of CoA in mouse oocytes.

## 3 Discussion

Binding partners and post-translational modifications regulate the pleiotropic cellular functions of Aurora A by switching it between inactive and active conformations [74]. The intrinsic catalytic activity of Aurora A is low prior to mitosis, and activation involves autophosphorylation of Thr 288 in the activation loop, which is promoted through interaction with microtubule-associated proteins such as TPX2 and TACC3. In this study, we identify CoA as a novel factor that binds and inhibits Aurora A activity *in vitro* and in human cells, potentially as part of the cellular response to oxidative stress. Aurora A CoAlation was not observed in exponentially growing cells, but was significantly induced after oxidative stresses. Consistently, microinjection of CoA into mouse oocytes induced cell cycle effects, which included atypical chromosome alignment and a failure to build a metaphase II spindle. This phenotype is consistent with the chemical or genetic ablation of Aurora A activity [12]. Mechanistically, Aurora A CoAlation is initiated by anchoring the ADP moiety of CoA to the ATP-binding pocket of a partially oxidised form of Aurora A, which involves a selectivity-determining interaction between the 3′-phosphate ADP moiety of CoA and Thr 217. Wedging of the ADP moiety then positions the flexible pantetheine tail of CoA with the thiol group in close proximity for covalent bond formation with Cys 290 in the Aurora A activation segment, two residues C-terminal from the regulatory phosphorylated Thr 288 residue. We speculate that under oxidative stress, covalent binding of the pantetheine thiol to Cys 290 keeps the ADP moiety firmly anchored to the ATP binding pocket, making it inaccessible to ATP, and preventing phosphotransferase activity. This “dual anchor” mechanism locks the kinase in an inactive state, potentially making CoA a more potent and selective endogenous inhibitor of Aurora A when compared to Aurora B or other related protein kinases.

### 3.1 What is the biological function of Aurora A CoAlation?

In addition to an inhibitory effect on Aurora A activity, CoAlation at Cys 290 may serve other regulatory purposes. First, CoA modification might protect Cys 290 from over-oxidation, which may lead to an irreversible loss of function and subsequent inactivation and/or degradation of Aurora A. The widespread ability of CoA to act as a low-molecular-weight antioxidant in response to oxidative and metabolic stress in prokaryotic and eukaryotic cells has been recently demonstrated [50,52]. In these studies, *in vitro* CoAlation of the catalytic Cys 151 in glyceraldehyde-3-phosphate dehydrogenase (GAPDH) was shown to protect this essential glycolytic enzyme against irreversible over-oxidation and the associated loss of activity. It remains to be seen whether oxidation of Cys 290 in Aurora A to sulfenic acid (which is reversible) facilitates covalent modification by CoA via a disulfide-exchange mechanism, protecting this highly susceptible thiol from irreversible over-oxidation. Secondly, covalent modification of Cys 290 by CoA may modulate the phosphorylation status of Thr 288, which is critical for Aurora A catalytic activity. Previous studies reported differential sensitivity of Thr 288 to dephosphorylation by phosphatase complexes of the phosphoprotein phosphatase (PPP) family. An intriguing possibility is that redox-induced covalent linkage of CoA to Cys 290, which is directly adjacent to Thr 288, may prevent the binding of phosphatases that control the dephosphorylation of Thr 288. In support of this hypothesis, we found that phosphorylation of Aurora A at Thr 288 was significantly increased under oxidative stress induced by diamide, H_2_O_2_, menadione and PAO. Alternative explanations for the observed accumulation of phosphorylated Aurora A are possible. CoA may act as a more efficient inhibitor of this form of the kinase or CoA modulates the activity of Aurora A regulatory phosphatases. Additionally, CoAlation of Aurora A might change recognition of the protein by the Thr 288 phosphospecific antibody and make it more readily detectable.

An involvement of CoA, a fundamental metabolic integrator in the regulation of Aurora A, provides new insight into our understanding of regulatory mechanisms of cell cycle progression mediated by metabolic adaptation to oxidative stress. Energy flow and biosynthetic processes are tightly regulated during the cell cycle. CoA plays a critical role in generating a diverse range of metabolically active thioester derivatives (including Acetyl-CoA, HMG-CoA, Malonyl-CoA and Acyl-CoA), which function as driving forces for generating ATP, the biosynthesis of macromolecules and the regulation of gene expression *via* protein acetylation. The presence of a highly reactive thiol group in CoA and its potential involvement in redox regulation has intrigued researchers for many decades, but progress in this field of study has been hampered by a lack of precise research tools and methodologies. Our findings are consistent with novel functions of CoA in cellular response to oxidative and metabolic stress, which involves covalent attachment of this coenzyme to redox-sensitive Cys residues [50]. Protein CoAlation is therefore emerging as an important post-translational modification implicated in redox regulation of a diverse range of proteins [44], including redox-controlled protein kinases [75].

Our work also demonstrates a hitherto unappreciated CoAlation of Aurora A during cellular response to oxidative stress. This mechanism of regulation may thus be relevant to other members of the human kinome that possess Cys residues in the activation segment, or elsewhere [76,77]. This includes other basophilic Ser/Thr kinases such as PKA, although PKA inhibition by CoA was not detected *in vitro*. However, the reversible glutathionylation of Cys 199, equivalent to Cys 290 in Aurora A, has previously been demonstrated [78], and correlates with functional Cys 199 redox status in controlling T-loop phosphorylation at the adjacent Thr 197 site in PKA, equivalent to Thr 288 in Aurora A [79,80]. In addition to Aurora A, CoA also modestly inhibited several unrelated protein kinases, including SRC, YES, BTK and FGFR1 (Table 1). However, none of these tyrosine kinases contain a Cys residue in the activation segment. It remains to be observed whether covalent modification of tyrosine kinases by CoA occurs in cells, and if so, whether it is also induced by oxidative stress. In the case of CoA, our data suggest that the “dual anchor” inhibitory mechanism in Aurora A permits precise spatial communication between the ATP-binding site and the activation loop, explaining the very high degree of specificity for CoAlation of Aurora A and preventing a high-affinity interaction even towards very closely-related kinases, such as Aurora B.

## 4 Conclusion

Our findings raise the interesting possibility that new covalent approaches can be developed to target Aurora A, or other kinases possessing Cys residues in the activation segment, with small inhibitory molecules. This dual targeting mechanism might be the basis of a new approach for design of specific, and potentially irreversible, Aurora A small molecule inhibitors, with long-time target engagement in cells. The majority of protein kinase (including Aurora A) inhibitors are currently ATP competitive (non-covalent) inhibitors, which are designed to gain at-least some selectivity through the recognition of unique features of conformation-specific ATP-binding pockets. However, there has been a resurgence of interest in generating covalent compounds targeting Cys residues in drug targets, most notably RAS [81], previously considered undruggable. Moreover, biochemical tool compounds [82,83] and clinical inhibitors covalently targeting oncogenic kinase, are enjoying significant clinical success [84]. However, one of the liabilities of such compounds is the appearance of drug resistance, caused by point mutations in the Cys residue at the covalent drug interface. In this study, we report a new ‘dual-mode’ of Aurora A inhibition by the metabolic integrator CoA, which also reveals how specific targeting of kinases might be accomplished with a distinct class of irreversible inhibitor, whose ability to covalently trap kinases in an inhibitory conformation could represent a new type of redox-mediated approach to tuning signaling outputs.

## 5 Materials and methods

### 5.1 Reagents and chemicals

The generation and characterization of the monoclonal anti-CoA antibody 1F10 has been described previously [50]. All common chemicals and biochemicals were obtained from Sigma-Aldrich unless otherwise stated, including CoA, dpCoA, dsCoA, ATP, and ADP. The following antibodies were employed: mouse anti-CoA antibody; mouse anti-FLAG M2 antibody (Sigma-Aldrich); rabbit anti-Aurora A and anti-Aurora B antibodies (Merck-Millipore); rabbit anti-pT288 Aurora A (Cell Signaling Technology), Alexa Fluor 680 goat anti-mouse IgG H&L (Life Technologies) and IRdye 800 CW goat anti-rabbit IgG H&L (LI-COR Biosciences).

### **5.2** Kinase profiling screen and Aurora A IC_50_ determination

Profiling of 117 protein kinases with CoA, dpCoA and ADP was carried out at the International Centre for Protein Kinase Profiling at the University of Dundee. Each compound was tested *in vitro*, in duplicate, at 100 μM final concentration using recombinant kinases, model substrates and optimal concentrations of ATP. Bacterially expressed full-length His-Aurora A was used to determine the IC_50_ value for CoA (in the presence or absence of DTT), dpCoA, ADP and desulfo-CoA. The assay was performed in the presence of 5 μM competing ATP. To examine whether the inhibitory effect of CoA on Aurora A is modulated by TPX2, IC_50_ values for CoA were also generated in the presence and absence of 1 μg TPX2 (residues 1–43) fragment.

### 5.3 *In vitro* Aurora A kinase assay

Aurora A kinase activity was assayed by measuring incorporation of γ^33^P-ATP into myelin basic protein (Sigma). Purified recombinant protein (100 ng) was incubated at room temperature for 30 min in a total volume of 15 μl containing 50 mM HEPES pH 7.5, 10 mM MgCl_2_, 1 mM EGTA, 0.05% Brij-35, 0.5 mg/ml myelin basic protein, and 5 μM γ^33^P-ATP (100–10,000 dpm/pmol). The reaction was stopped by spotting the reaction mixture onto squares of P81 phosphocellulose ion exchange paper (Whatmann), which were then immersed in 1.5% (v/v) phosphoric acid. After washing twice in 1% phosphoric acid followed by two washes in distilled water, the papers were air dried and radioactivity was counted by a scintillation counter and recorded by Quantasmart version 2.03.

### **5.4** *In vitro* CoAlation assay

For CoAlation assays, 0.5 μg of purified recombinant preparations of wild type and mutant forms of His-Aurora A (Cys290Ala; Cys393Ala, Cys290/393Ala, Thr217Glu) and His-Aurora B (Glu161Thr) were incubated with a mixture of oxidised and reduced forms of CoA (CoASH and CoASSCoA, 1 mM final) in 20 mM Tris-HCl, pH 7.5 for 30 min at RT. The reaction was stopped by adding SDS gel-loading buffer without DTT, but containing 10 mM NEM. To examine whether binding of the 3′-phosphate ADP moiety of CoA to the Aurora A ATP binding-pocket co-ordinates the pantetheine thiol for disulfide bond formation with Cys 290, the *in vitro* CoAlation assay was carried out with 100 μM CoA and increasing concentrations of ATP (0–10 mM).

### 5.5 CoA Sepharose pull-down assay

Exponentially growing HepG2 cells were lysed on ice in buffer, containing 50 mM Tris-HCl pH 7.5, 150 mM NaCl, 5 mM ethylenediaminetetraacetic acid (EDTA), 50 mM sodium fluoride (NaF), 5 mM tetra-sodium pyrophosphate (Na_4_P_2_O_7_) and 1% (v/v) Triton X-100, supplemented with 1x Protease Inhibitor Cocktail (PIC, Roche). After centrifugation, the supernatant was incubated on a rotating wheel with CoA Sepharose or Sepharose alone for 2h at 4 °C. Beads were then washed extensively with lysis buffer and bound proteins were eluted from beads with 2x SDS loading buffer. To elute proteins that bound specifically to CoA Sepharose, the cell lysis buffer was supplemented with 100 μM CoA (CoA eluted fraction). Eluted proteins were separated by SDS-PAGE and Western blotted with anti-Aurora A antibody.

### 5.6 Lanthascreen Eu Kinase FRET binding assay

A Lanthascreen Eu Kinase FRET Binding Assay (Invitrogen) was employed to determine IC_50_ values for the interaction of Aurora A with CoA, dpCoA, ATP, and ADP. In this assay, we used bacterially expressed His-Aurora A in active and inactive (PP1-treated, pT288 dephosphorylated) states. Recombinant Aurora A was incubated with Europium-conjugated anti-histidine tag antibody and an Alexa Fluor 647-labelled tracer, which binds to the ATP binding pocket of Aurora A. The close proximity of the anti-histidine (epitope-tag) antibody and the tracer results in a high degree of FRET (fluorescence resonance energy transfer) from the europium donor fluorophore to the Alexa Fluor 647 acceptor fluorophore. ATP-competitive inhibitors, such as CoA, displace the tracer from the active site, causing a loss of FRET signal. Assay set-up was performed as described by the manufacturer. Briefly, the time-resolved fluorescence resonance energy transfer assay (TR-FRET) was performed in black, low volume 384 well plates (Corning). Each well contained 5 nM kinase, 2 nM Eu-anti-His antibody and 10 nM kinase tracer 236 in kinase buffer A (50 mM Hepes pH 7.5, 10 mM MgCl_2_, 1 mM EDTA, 0.01% (v/v) Brij-35), varying amounts of CoA, dpCoA, ATP, and ADP. The reaction mix was incubated for 1h at room temperature. The signal was measured at 665/620 nm emission ratio using BMG LABTECH plate reader (PHERAstar). All assays were performed using duplicates. The 11-point response curves were generated using GraphPad Prism software from the inhibition data generated.

### 5.7 Plasmids and protein purification for differential scanning fluorimetry (DSF)

For enzyme and DSF assays, full-length human Aurora A and Aurora B and 1–43 TPX2 were produced in BL21 (DE3) pLysS *E. coli* cells (Novagen) with expression induced with 0.5 mM IPTG for 18h at 18 °C and purified as *N*-terminal 6His-tag fusion proteins by immobilized metal affinity chromatography (IMAC) and size exclusion chromatography using a HiLoad 16/600 Superdex 200 column (GE Healthcare) equilibrated in 50 mM Tris/HCl, pH 7.4, 100 mM NaCl, 10 % (v/v) glycerol and 1 mM DTT. Asp274Asn and Thr217Glu Aurora A mutants were generated by PCR-site directed mutagenesis, expressed and purified as described previously [40]. Cys290Ala Aurora A was generated using standard procedures, and purified as described.

### 5.8 Differential scanning fluorimetry

Thermal-shift assays were performed with a StepOnePlus Real-Time PCR machine (Life Technologies) using Sypro-Orange dye (Invitrogen) and thermal ramping (0.3 °C in step intervals between 25 and 94 °C). All proteins were diluted to a final concentration of 5 μM in 50 mM Tris/HCl, pH 7.4 and 100 mM NaCl in the presence or absence of the indicated concentrations of ligand (final DMSO concentration no higher than 4 % v/v). CoA, dephosphoCoA or kinase inhibitors diluted from 10 mM DMSO stocks [59] were assayed as described previously [60]. Normalized data were processed using the Boltzmann equation to generate sigmoidal denaturation curves, and average T_m_/ΔT_m_ values calculated as previously described [61] using GraphPad Prism software.

### 5.9 Molecular docking

Docking of CoA in the crystal structure of Aurora A kinase (PDB_ID: 1OL7) [10] was carried out using CDOCKER (Discovery Studio 3.1, Accelrys Inc). In the crystal structure, a 7 Å grid was selected around the ATP binding site in order to define the location of probable CoA binding site. Twenty-five conformations of CoA were generated and ten docking poses were analysed.

### 5.10 Crystallization and structure determination of the Aurora A 122–403/CoA complex

Aurora A (amino acids 122–403) was produced as described in earlier work [62]. The kinase was subject to size exclusion chromatography on a HiLoad 16/600 Superdex 200 pg column (GE Healthcare) equilibrated in 20 mM Tris pH 7.0, 0.2 M NaCl, 5 mM MgCl_2_, 10 % (v/v) glycerol prior to crystallization trials. Aurora A was concentrated to 16.5 mg/ml and incubated with 5 mM CoA for 30 min at 30 °C. Crystallization screens were laid down in 96-well MRC plates using a Mosquito LCP crystallization robot (TTP Labtech) and incubated at 295 K. Crystals were produced in 0.03 M MgCl_2_·6H_2_0, 0.03 M CaCl_2_·2H_2_0, 0.1 M MOPS/HEPES-Na pH 7.5, 20 % (v/v) PEG 500 MME, 10 % (w/v) PEG 20 000 (Molecular Dimensions) and flash frozen directly from the drop.

X-ray diffraction data were collected on beamline I03 at Diamond Light Source, Oxford, England from a single crystal. Data processing was performed by the *xia2 3dii* automated data-reduction platform at Diamond [63]. The structure of the complex was solved by molecular replacement using Phaser-MR [64] and the structure of Aurora A 122–403 C290A, C393A (PDB 4CEG) as a model [65]. Phenix.refine was used to solve the structure and carry out iterative refinement [66]. Model building was performed using Coot [67]. Structure validation and data quality were determined by Molprobity [68].

### 5.11 Human cell culture

HEK293 and HEK293/Pank1β cells were cultured in Dulbecco's Modified Eagle Medium (DMEM) (Lonza) supplemented with 10% foetal bovine serum (FBS) (Hyclone), 50 U/ml penicillin and 0.25 μg/ml streptomycin (Lonza). HepG2 cells were cultured in Williams E media (Lonza) supplemented with 10 % FBS, 50 U/ml penicillin and 0.25 μg/ml streptomycin. All cell lines were tested and shown to be free of mycoplasma infection. Generation of HEK293/Pank1β cell line with stable overexpression of Pantothenate kinase 1β was previously reported [50].

### 5.12 Transient transfection of HEK293/PANK1β cells and treatment with oxidising agents

HEK293/Pank1β cells were transfected at ~60% confluence with pCMV6-Entry/FLAG-Aurora A and pCMV6-Entry/FLAG-Aurora B plasmids, according to the manufacturer's protocol using Turbofect reagent (Thermo Scientific). Transfected cells were allowed to grow for 24 h in complete DMEM with 10 % FBS. The medium was replaced with pyruvate-free DMEM supplemented with 5 mM glucose and 10 % FBS and cells were incubated for another 24 h. Pyruvate was removed from the media because it can act as an antioxidant and inactivate ROS. Cells were then treated with H_2_O_2_ (250 μM), menadione (50 μM), phenylarsine oxide (PAO, 10 μM), diamide (500 μM) or rotenone (1  μM) for 30 min at 37 °C in pyruvate-free DMEM supplemented with 5 mM glucose. Cells were harvested by pressure washing and centrifuged at 1800 g for 5 min at RT.

### 5.13 Cell lysis, immunoprecipitation and western blot analysis

Harvested cells were lysed at 4 °C for 20 min in lysis buffer, containing 50 mM Tris-HCl pH 7.5, 150 mM NaCl, 5 mM EDTA, 50 mM NaF, 5 mM Na_4_P_2_O_7_ and 1 % Triton X-100, supplemented with fresh 100 mM NEM and fresh 1x PIC. Total cell lysates were centrifuged at 20817*×g* for 10 min at 4 °C and the supernatant was collected for further analysis. Protein concentration was measured using the Bicinchoninic acid Protein Assay Kit (Thermo Scientific). Immunoprecipitation of transiently expressed FLAG-Aurora A and FLAG-Aurora B from cell lysates was carried out using Protein G Sepharose (Generon) and anti-FLAG antibody (Sigma-Aldrich). Proteins were eluted from beads with 2x SDS loading buffer and separated by SDS-PAGE under non-reducing conditions. Resolved proteins were transferred to a PVDF membrane (Bio-Rad Laboratories), which was then blocked with Odyssey blocking buffer. The membrane was incubated in anti-CoA or anti-FLAG antibodies for 2 h at room temperature (RT) or overnight at 4 °C, and then with secondary antibodies for 30 min (RT). Immunoreactive bands were visualised using Odyssey Scanner CLx and Image Studio Lite software (LI-COR Biosciences).

### 5.14 Microinjection of mouse oocytes

Mouse oocytes arrested at the GV stage were injected with different concentrations of CoA (0.15–5 mM). Since the commercial preparation of CoA used for microinjection contained 3 mol of lithium ions per mole of CoA, control oocytes were injected with an equivalent amount of LiCl. In a separate study, oocytes were injected with 3 mM CoA, ADP, AMP, 3′-5′-ADP and pantetheine. To examine the effect of injected compounds on spindle formation, oocytes were fixed 17 h after GV release and stained for alpha-tubulin (green) and DNA (blue).

### 5.15 Intact mass spectrometry

To generate CoA or dPCoA complexes with Aurora A, 10 μM WT Aurora A was incubated with 100 μM CoA or dpCoA for 15 min at room temperature. To evaluate the interaction, intact complexes were desalted using a C4 desalting trap (Waters MassPREP™ Micro desalting column, 2.1 × 5 mm, 20 μm particle size, 1000 Å pore size). Aurora A was eluted with 50 % (v/v) MeCN, 0.1 % (v/v) formic acid. Intact mass analysis was performed using a Waters nano ACQUITY Ultra Performance liquid chromatography (UPLC) system coupled to a Waters SYNAPT G2, as described [60]. Samples were eluted from a C4 trap column at a flow rate of 10 μL/min using three repeated 0–100 % acetonitrile gradients. Data was collected between 400 and 3500 *m/z* and processed using MaxEnt1 (maximum entropy software, Waters Corporation).

### 5.16 Ion mobility

IM-MS analysis was performed on a Waters Synapt G2-S*i* instrument. Purified Aurora A was buffer exchanged into 50 mM NH_4_OAc (LC grade, Sigma) as previously described [69]. Typically, 1–3 μl of 2–5 μM sample was analysed using borosilicate emitters (Thermo ES 387). Spraying voltage was adjusted to 1.1–1.8 kV, sampling cone was 20 V. Pressure in the travelling wave (T-wave) ion mobility cell was 2.78 mbar (nitrogen), wave height was kept at 30 V, wave velocity at 750 m/s. In order to experimentally determine collision cross section (CCS), drift time through the T-wave mobility cell was performed using β-lactoglobulin A (Sigma L7880), avidin (Sigma A9275), transthyretin (Sigma P1742), concanavalin A (Sigma C2010) and serum albumin (Sigma P7656) according to standard protocols. The exact hard sphere scattering (EHSS) model implemented in the Mobcal software was used to calculate CCS values on the basis of X-ray structures, as described previously [69].

### 5.17 Mass spectrometry and data processing

*In vitro* CoAlated His-Aurora A or immunoprecipitated FLAG-Aurora A were digested with sequencing grade trypsin (Promega). After heat-inactivation of trypsin, CoAlated peptides were immunoprecipitated with anti-CoA antibody cross-linked to Protein G Sepharose. Immunoprecipitated peptides were treated with Nudix 7 and enriched further by an IMAC column before LC–MS/MS analysis. The resulting samples were analysed by nano-scale capillary LC-MS/MS using an Ultimate U3000 UPLC System (Dionex) fitted with a 100 μm × 2 cm PepMap100 C18 nano trap column and a 75 μm × 25 cm PepMap100 C18 nano analytical column (Dionex). Peptides were eluted using an acetonitrile gradient and sprayed directly via a nano-flow electrospray ionization source into the mass spectrometer (Orbitrap Velos, Thermo Scientific). The mass spectrometer was operated in data dependent mode, using a full scan (*m*/*z* = 350–1600) in the Orbitrap analyser, with a resolution of 60 000 at m/z = 400, followed by MS/MS acquisitions of the 20 most intense ions in the LTQ Velos ion trap. Maximum FTMS scan accumulation times were set at 250 ms and maximum ion trap MSn scan accumulation times were set at 200 ms. The Orbitrap measurements were internally calibrated using the lock mass of polydimethylcyclosiloxane at *m*/*z* 445.120025. Dynamic exclusion was set for 30 s with exclusion list of 500. LC-MS/MS raw data files were processed as standard samples using MaxQuant [70] version 1.5.2.8, which incorporates the Andromeda search engine. MaxQuant processed data was searched against Human UniProt protein database. Carbamidomethyl cysteine, Acetyl N-terminal, N-ethylmaleimide cysteine, oxidation of methionines, CoAlation of cysteine with delta mass 338, 356 and 765, were set as variable modifications. For all data sets, the default parameters in MaxQuant were used, except MS/MS tolerance, which was set at 0.6 Da and the second peptide ID was unselected.

## CRediT authorship contribution statement

This study was originally conceived by I.G. Y.T., A.Z., J.B., Y·H., T.T., F.B. and D.P.B. performed aspects of cell biology, biochemical and biophysical assays; S.G.B. and R.B. designed and carried out crystallization, structure determination and analysis of the Aurora A/CoA complex; M. V. and S.F. performed ion mobility and intact mass spectrometry analysis; E.C. and L.G. carried out molecular docking studies; O.G. and V.F. developed and characterised anti-CoA Mabs; J.B. carried out mouse oocytes microinjection experiments; S·Y·P–C. and M.S. designed and performed the LC-MS/MS experiments; Y.T., P.A.E., R.B., C.E.E., J.C., A.T. and I.G. designed and analysed experiments; I.G., P.A.E. and R.B. wrote the manuscript with the assistance and approval of all authors.

## Funding sources

This work was funded by grants to I.G. (UCLB 13-014 and 11-018; Rosetrees Trust CM239-F2; BBSRC BB/L010410/1 and BB/S009027/1); P.A.E, (North West Cancer Research Fund CR1088/1097), R.B. (Cancer Research UK C24461/A23302); C.E.E. (BBSRC BB/M012557/1 and BBSRC DTP studentship to S·F); and V.F. (National Academy of Sciences of Ukraine 0110U000692); T.T. was funded by UCL ORS and UCL GRS awards.

## Conflicts of interest

The Authors declare that there are no competing interests associated with the manuscript.
