# Supplementary material for: Covalent Aurora A regulation by the metabolic integrator coenzyme A
Source: Redox Biol. 2019 Sep 5;28:101318. doi: 10.1016/j.redox.2019.101318 (PMC6812009; doi:10.1016/j.redox.2019.101318)
Supplement: Multimedia component 2 [file mmc2.docx]

**Supplemental Information**

**Supplementary Figure 1. Binding of endogenous cellular Aurora A to CoA.** CoA Sepharose or Sepharose control beads were employed in an immunoprecipitation assay using extracts prepared from exponentially growing HepG2 cells. Bound proteins were eluted from beads with 100 μM CoA (CoA-eluted fraction) or 2x SDS loading buffer. Eluted proteins were separated by SDS-PAGE and immunoblotted with anti-Aurora A antibody.

**Supplementary Figure 2. Molecular docking of CoA to Aurora A.** Model of CoA bound to Aurora A (PDB 1OL7). The location of Thr 217 adjacent to the 3’phospho-adenine of CoA, and Cys 290 in the kinase activation segment, are both indicated.

**Supplementary Figure 3. MS analysis of T288 phosphorylated and CoAlated Aurora A.** LC–MS/MS spectra of Aurora A tryptic peptides derived from **(A)** *in vitro* CoAlation or **(B)** FLAG-Aurora A isolated from peroxide-treated cells, where Aurora A is CoAlated on Cys290. The ion chromatogram for peptide with m/z of 1183.53 is shown, CID confirmed Cys290 as the site of CoAlation.

**Supplementary Figure 4. Brief exposure to hydrogen peroxide induces *in vitro* dimerization of wild type Aurora A, but not the C290A mutant.** 0.5 μg of purified phosphorylated human Aurora A protein (WT or C290A) was incubated in the presence or absence of 1 mM H2O2 for 15 minutes, and Aurora A proteins were boiled in SDS sample buffer in the presence (+DTT, reduced) or absence (-DTT) of reducing agent, prior to SDS-PAGE and immunoblotting. The migration pattern of Aurora A was visualised with an anti-6His antibody, and two different exposures are shown for comparison. The putative oxidised Aurora A dimer is absent in the C290A preparation.
